# Resorption of retromolar bone grafts after alveolar ridge augmentation—volumetric changes after 12 months assessed by CBCT analysis

**DOI:** 10.1186/s40729-020-00285-9

**Published:** 2021-01-21

**Authors:** Andres Stricker, Reinhilde Jacobs, Frederik Maes, Tabea Fluegge, Kirstin Vach, Jonathan Fleiner

**Affiliations:** 1 Center of Implantology, Periodontology and 3D Head-and-Neck Imaging, Konstanz, Germany; 2 Department of Oral and Maxillofacial Surgery, Medical Center, University of Freiburg, Freiburg, Germany; 3 Faculty of Medicine, University of Freiburg, Freiburg, Germany; 4 OMFS-IMPATH Research Group, Department of Imaging and Pathology, Faculty of Medicine, University of Leuven, Leuven, Belgium; 5 Department of Oral and Maxillofacial Surgery, University Hospitals Leuven, Leuven, Belgium; 6 Department of Dental Medicine, Karolinska Institutet, Stockholm, Sweden; 7 ESAT/PSI & Medical Imaging Research Center, Faculty of Engineering Sciences, University of Leuven, Leuven, Belgium; 8 Department of Oral and Maxillofacial Surgery, Charité University of Medicine Berlin, Campus Benjamin Franklin, Berlin, Germany; 9 Institute of Medical Biometry and Statistics, Faculty of Medicine and Medical Center, University of Freiburg, Freiburg, Germany

**Keywords:** Ridge augmentation, Bone remodeling, Bone resorption, Onlay graft, CBCT, Three-dimensional imaging, Alveolar bone

## Abstract

In this pilot study, a volumetric analysis of retromolar onlay bone grafts over a period of 12 months was conducted, using repeated CBCT imaging combined with automated image registration.

Eleven patients being treated with 16 bone grafts taken from the retromolar area were examined by CBCT scanning prior to bone augmentation (T0), immediately after bone augmentation (T1) and after a healing time of 12 months after augmentation (T2). Graft volumes were measured at each time point after automated image registration of consecutive CBCT scans.

The mean volume of the augmented site was 372.2 ± 179.4 mm^3^. Resorption relative to the original augmented volume was 43.7% ± 19.0% after 12 months.

Three-dimensional graft resorption could be precisely depicted by the use of automated image registration for CBCT data over a period of 12 months and demonstrated extensive volumetric changes of bone grafts taken from the ascending ramus of the mandible.

Graft resorption and continuous bony remodeling of the grafted site before and after implant insertion have to be carefully considered by the clinician.

**Supplementary Information:**

The online version contains supplementary material available at 10.1186/s40729-020-00285-9.

## Introduction

Sufficient bone quantity and quality at the recipient site is a major prerequisite for long-term success of dental implants [1–3]. However, in many situations, there is a bone deficiency, indicating a surgical procedure that predictably leads to sufficient bone quantity prior to implant insertion [4–6].

Guided bone regeneration (GBR), alveolar distraction osteogenesis, and onlay grafting have been described to augment the horizontal and vertical bone volume [7–10]. Transplantation of autogenous bone grafts is the standard procedure for reconstruction of a severely resorbed alveolar crest prior to implant insertion [11–19].

However, bone resorption both in height and width has been described for onlay grafts harvested from the iliac crest [20–24] and ascending ramus of the mandible [25–28].

Mechanical calipers and different radiographic techniques have been applied for assessment of the augmented areas prior to implant placement [25, 28]. Conventional radiographic imaging, using the parallel technique, bitewings or panoramic X-rays, allows an estimation of the vertical dimension of the bone graft. However, due to the inevitable overlay of anatomical structures and the presence of image distortion and blurring, adequate information about the horizontal dimension and the three-dimensional volume changes cannot be derived [29, 30]. In contrast, computed tomography (CT) and cone beam computed tomography (CBCT) technology may overcome these fundamental limitations and may be used for accurate three-dimensional representation of the alveolar bone before and after bone augmentation. Volumetric CT studies of autogenous onlay grafts harvested from the iliac crest demonstrated a rapid initial loss of bone height during the first 6 months of healing [22, 31].

Three-dimensional analysis using CBCT got increasingly popular because of its lower radiation and higher resolution compared to volumetric CT [32]. Here, long-term stability of implants inserted in the pristine bone [33–35] and assessment of onlay bone graft have been studied [36–39].

In order to enable adequate prediction of volumetric changes of autogenous onlay bone grafts over time, the aim of this pilot study was to conduct a volumetric analysis using CBCT imaging at different postoperative time points combined with an automated image registration procedure [40], to accurately evaluate volume alterations of onlay graft augmentation with autogenous bone taken from the ascending ramus of the mandible over a period of 12 months.

## Materials and methods

### Patient selection

In this study, patients of at least 18 years with autogenous bone grafts from the ascending alveolar ramus prior to dental implant placement were considered for this retrospective analysis. Study approval was obtained from the Ethical Committee of the University Medical Center Freiburg, Germany (138/14).

All patients were healthy, non-smokers and had no general contraindications as history of malignancy, antiresorptive-, radio- or chemotherapy, pregnancy or nursing, and general diseases which may negatively affect bone or connective tissue metabolism or bone turnover rate.

Local inclusion factors comprised a transversal width of the alveolar crest < 3 mm and a vertical height > 7 mm.

In addition, for retrospective inclusion in the study independently of study aspects, CBCT scans must have been acquired at different time points:

- Prior to bone augmentation (T0)
- Immediately after bone augmentation (T1))
- 12 months after bone augmentation (T2).

A total of 220 patients being treated with retromolar bone grafts were initially screened for inclusion in this study. Based on the criteria above, 11 patients (10 female, 1 male) having a mean age of 53.0 years (range 20–69 years) could be selected for further analysis.

### Surgical procedure

Bone augmentation was performed with local anesthesia in 8 patients, while 3 patients were operated under general anesthesia.

After crestal incision and elevation of a muco-periostal flap, the atrophy of the recipient site was evaluated. Thereafter, a paramarginal incision in the retromolar region of the mandible was performed to get access for harvesting the cortico-cancellous bone graft from the ascending ramus of the mandible using a piezotome and chisel.

Bone grafts were adapted to atrophic ridge anatomy with diamond burs before fixation to the recipient bone by two titanium screws of 1.5 mm diameter and 8 mm length (Bone Fixation Aesculap, Tuttlingen, Germany). Periostal release incision was followed by tension free wound closure with non-resorbable sutures (Seralon 5.0, Serag Wiessner, Germany). After surgery, antibiotics (Clindamycin 600 mg, 3×/d, 7 days), pain medication (Ibuprofen 400), and rinsing irrigation (0.1% CHX rinsing solution) were administered.

### CBCT analysis

CBCT scans acquired at different time points were evaluated to assess volumetric changes of onlay grafts over time. CBCT scanning was performed prior to bone augmentation (T0), immediately after bone augmentation (T1), and at 12 months after bone augmentation (T2).

All CBCT scans were acquired with an iCat Cone Beam 3D scanner (Imaging Sciences International, LLC, Hatfield, PA, USA) with the following scan parameters: 0.3 mm voxel size, 5 mA, 120 kV, and scanning time 8.9 s.

For each patient, the baseline scan (T0) was aligned to the post-op scan (T1) and the follow-up scan (T2,) using an automated image registration procedure [40]. A rigid registration was used that was restricted to either the mandible or the maxilla, depending on the implant site. The accuracy of the registration was visually verified for all cases. In this way, identic orientation of the anatomic details of the region of interest (ROI) could be guaranteed.

**Fig. 1:**
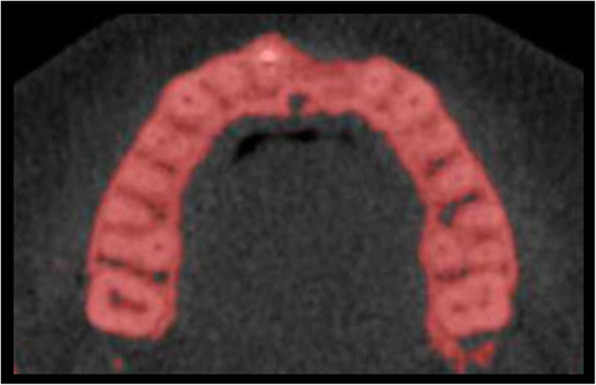
CBCT scan (axial view) of lower jaw at time point T0 before onlay graft

**Fig. 2:**
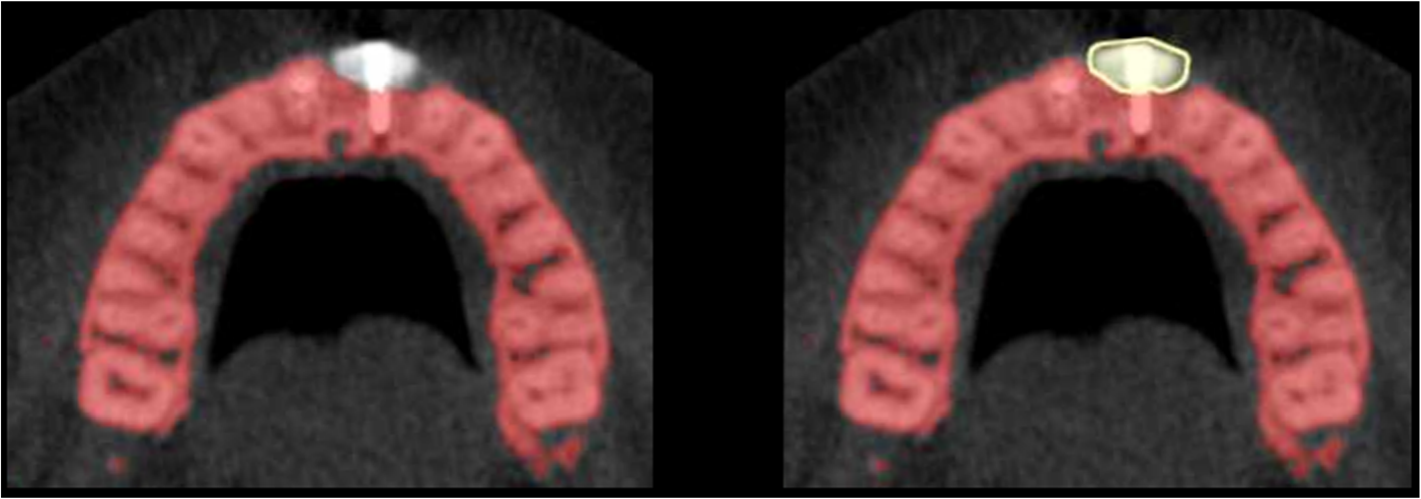
CBCT scan (axial view) of lower jaw at time point T1 postoperatively after onlay graft

**Fig. 3:**
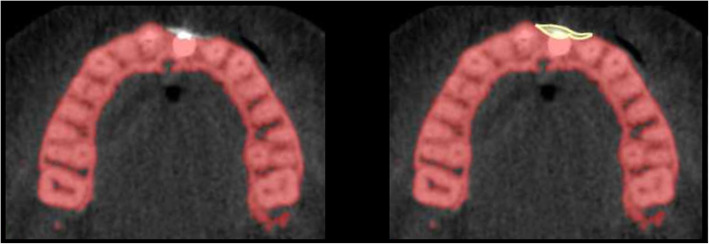
CBCT scan (axial view) of lower jaw at time point T2, 12 months after onlay graft

In a next step, the bony contours of the ROI for T0 were delineated and visualized against every aligned follow-up scan (T1, T2) to ensure bone volume measurements within identical anatomical regions at all time points (Figs. 1, 2, and 3). The additional amount of grafted bone extending the defined contours of the T0 region was measured by manual slice-by-slice delineation in the axial CBCT images (Figs. 1, 2, and 3). In vertical direction, axial CBCT slices between the marginal alveolar bone crest and the level of the maximal extent of the onlay graft were considered [41]. Consequently, the total volume of the augmented site was calculated by automated interpolation between the axial slices (MeVisLab V.1, MeVis Medical Solutions AG, Bremen, Germany). All CBCT scans were analyzed by two independent examiners that were not involved in the surgical therapy of these patients.

### Statistical evaluation

For this study, all available patients with a CBCT scan 12 months after augmentation were used; hence, no sample size calculation was performed. For the 11 patients, an effect size of 2.3 can be detected.

For descriptive analysis, mean and standard deviation were computed. Intraclass correlation coefficients (ICC) were used to evaluate the agreement between the two observers for the different volume measurements. For further analysis, the mean volume values over the observers were used. A paired *t* test was applied to check for differences between the original bone volume and the 12-month value. Linear regression models with robust variance estimators were used to analyze both influence of jaw and age on the volume value.

All calculations were performed with the statistical software STATA 16 (StataCorp LT, College Station, TX, USA). The probability level for statistical significance was set to *P* < 0.05.

## Results

Surgical rehabilitation affected 11 patients having 16 bone grafts, 4 in the maxilla, and 12 in the mandible, respectively.

All onlay bone grafts healed uneventfully, without soft tissue dehiscence, and graft incorporation was successful to allow placement of 22 dental implants after a healing time of 104.4 ± 11.5 days.

Final prosthodontic rehabilitation by implant-supported restorations was performed 105.2 ± 17.7 days after implant placement or 209.6 ± 24.6 days after bone augmentation. No implants were lost during the entire observation period of 510.4 ± 180.2 days and thereafter up to now.

**Fig. 4:**
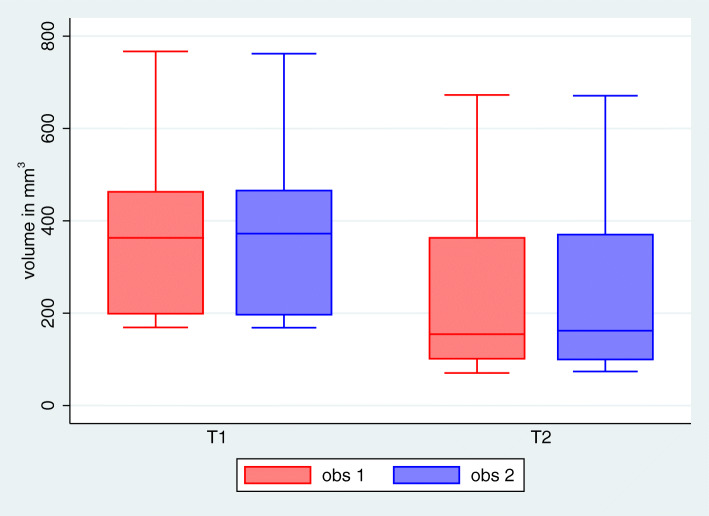
Mean bone volume within identical ROIs at different time points T1 and T2 after augmentation with onlay grafts harvested from the retromolar area

We observed a very high intraclass correlation between the volume measurements of the two observers for all time points of 0.999 (*p* < 0.0001). For further analyses, the mean volume measurements over the two observers were used (Fig. 4).

**Fig. 5:**
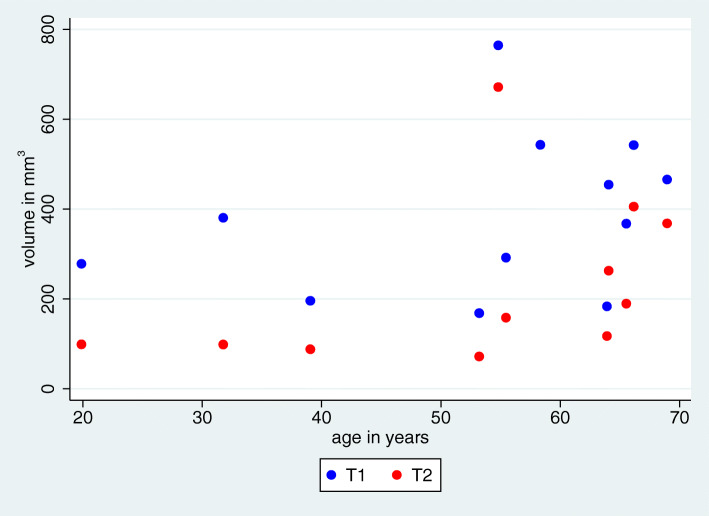
Relationship between age and bone volume at different time points T1 and T2 after augmentation

The mean bone volume of the augmented site was 372.2 ± 179.4 mm^3^ immediately post-operatively (T1) and 230.2 ± 185.7 mm^3^ after 12 months (T2), leading to an extent of total resorption relative to the original bone volume of 43.7% ± 19.0%, being highly significant (*p* < 0.001) (Figs. 4 and 5).

Furthermore, the influence of age and jaw on the volume change was analyzed. We observed a regression coefficient of − 1.73 for age, meaning that the volume change from baseline to the last time point became lower with increasing age, but was not significant (*p* = 0.175).

Localization of onlay graft augmentation demonstrated differences: a mean volume change from baseline to T2 in the lower jaw was 113.7 ± 34.7 mm^3^ (37.6% ± 16.7%). In the upper jaw, the difference was larger 217.7 ± 56.2 mm^3^ (60.2% ± 16.4%), being statistically significant (*p* = 0.01).

## Discussion

This pilot study is a three-dimensional CBCT study using an automated image registration procedure to analyze bone resorption of onlay grafts harvested from the ascending ramus of the mandible.

The use of cross-sectional information provided by CT scans to examine bone resorption of onlay grafts was introduced by Nyström et al. in 1995 [42]. The authors performed linear measurements of width and height of iliac onlay grafts in arbitrarily defined two-dimensional cross-sections oriented along the simultaneously inserted implants [42]. A comparable measurement protocol was followed by Malchiodi et al. to measure bone width in axial CT cross-sections of augmented maxillae and mandibles [26]. The previously described methods make use of CT technology to assess bone width in oro-vestibular direction; however, a volumetric assessment of bone grafts was not performed.

Anitua et al. and Monje et al. transferred linear measurement protocols to cross-sectional images derived from CBCT imaging and measured bone width of onlay grafts at defined levels above the residual alveolar bone [37, 39].

Volumetric measurement protocols using CT technology were mostly used to assess resorption of iliac onlay grafts [31, 43–45]. The outline of the grafted areas was manually delineated in multiple cross-sections of the complete onlay graft and added to obtain its volume. The orientation of cross-sections and the use of landmarks varied and authors did not disclose their protocol to differentiate between bone grafts and residual bone, especially after healing of bone grafts.

Spin-Neto et al. introduced the measurement of grafted and residual bone in each cross section through onlay bone grafts and documented bone resorption over the complete bone volume in each grafted area [46].

Lee and Kim obtained the volume of onlay grafts by assigning a range of gray values derived from the donor site of the mandible to grafted bone. However, no reference was found in their work regarding the accuracy of the selected volume and the consistency of standard gray values for bone grafts after transplantation and over the healing period [38].

Kloss et al. measured single site defects on CBCT scans in their height, width, and depth at the cervical level, the middle height of the defect, and at the apical level. All measurements were made on parasagittal sections perpendicular to the longitudinal axis of the adjacent teeth. Based on the radiographic measurements, the graft volume was inferred as the sum of the volumes of two superimposed four-sided rectangular frustums of pyramids [47].

When evaluating edentulous sites, precise identification of stable corresponding landmarks and defined borders within consecutive (CB-)CT scans is crucial for reliable assessment of bone changes at the augmentation site, especially as bone remodeling induces contour changes of the grafted bone block.

This study is based on automated image registration and geometric alignment of consecutive datasets for a more consistent and precise evaluation. As large variability of quantitative gray scale values (GSV) may occur in CBCT images due to various reasons (e.g., radiation dose, field size, scattered radiation, and limitations of applied reconstruction algorithms), the use of Hounsfield units for tissue characterization in CT may not be applied for CBCT [48]. As the use of quantitative GSV remains essential and cannot be avoided, our study was at least based on CBCT images acquired with identical exposure time and field-of-view to reduce GSV variability.

Furthermore, automated image registration and alignment of all images of the same patient allowed definition of identical ROI prior to evaluation of bone grafts in images of different time points. The residual bone was assessed before bone grafting using stable landmarks outside the ROI to obtain baseline values. Consecutive measurements of bone within the previously marked volume were subtracted by baseline values to obtain the actual volume of bone grafts over time.

Regarding the stability of onlay grafts after 12 months, so far, there is only a limited amount of studies available and data are controversial.

Sbordone et al. detected volume resorption between 35 and 51% in iliac crest transplants and 45% for onlay grafts from the chin [45]. In contrast, Kloss et al. stated on mandibular bone grafts in single tooth defects a shrinkage rate of 12.5% ± 7.8% after 12 months. This is in opposite to our present findings.

On the other hand, Lee and Kim showed a mean graft resorption of 25.4% at 5.5 months after coverage of onlay grafts from the ascending ramus of the mandible with particulate cortical bone mixed with fibrin sealant and a resorbable collagen membrane [36, 38].

The sparse and highly variable data documented for onlay bone grafts especially from the ascending ramus of the mandible do not allow to predict volume resorption during healing periods of three to six months before the placement of dental implants.

This pilot study showed a continuous three-dimensional bone resorption of autogenous onlay grafts of 43.3% at 12 months after bone augmentation, using a precise three-dimensional assessment based on automated image registration procedure. By adopting identical bony contours and anatomical borders within consecutive CBCT scans, potential manual measurement errors could be reduced.

Further studies with a clearly higher number of patients are necessary to document bone resorption after onlay grafting to establish standard values for clinical recommendations.

## Conclusions

CBCT imaging at different time points combined with an automated image registration procedure allowed to evaluate volume alterations of onlay graft augmentation over a period of 12 months, demonstrating in this study extensive volumetric changes of onlay bone grafts taken from the ascending ramus of the mandible.

Graft resorption and continuous bony remodeling of the grafted site before and after implant insertion have to be carefully considered by the clinician.

Randomized controlled studies with a larger study sample are needed to verify the present findings.

## Supplementary Information

**Additional file 1.**

## Data Availability

Please find all statistical data in the excel sheet enclosed in the supplemental material.
